# Influence of Obstructive Sleep Apnea on Oxidative Stress in Pregnancy

**DOI:** 10.3390/ijms26030886

**Published:** 2025-01-21

**Authors:** Laura Cànaves-Gómez, María Paloma Giménez Carrero, Ainhoa Álvarez Ruiz De Larrinaga, Andrés Sánchez Baron, Mercedes Codina Marcet, Amanda Iglesias Coma, Mónica De-La-Peña, María Concepción Piñas Cebrian, Susana García Fernández, José Antonio Peña Zarza, Daniel Morell-Garcia, Antonia Barceló Bennasar, Alberto Alonso-Fernández

**Affiliations:** 1 Institut d’Investigació Sanitària Illes Balears (IdISBa), 07120 Palma de Mallorca, Spain; laura.canaves@ssib.es (L.C.-G.); mariap.gimenez@ssib.es (M.P.G.C.); mercedes.codina@ssib.es (M.C.M.); amanda.iglesias@ssib.es (A.I.C.); monica.delapena@ssib.es (M.D.-L.-P.); m.c.pinas@ssib.es (M.C.P.C.); susana.garcia@ssib.es (S.G.F.); josea.pena@ssib.es (J.A.P.Z.); daniel.morell@ssib.es (D.M.-G.); antonia.barcelo@ssib.es (A.B.B.); 2 Sleep Unit, Hospital Universitario de Araba, 01009 Vitoria, Spain; ainoa.alvarezruizdelarrinaga@osakidetza.eus; 3 Instituto de Investigación BIOARABA, 01009 Vitoria, Spain; 4 Servicio de Neumología, Hospital Universitario Miguel Servet, 50009 Zaragoza, Spain; andr1944@separ.es; 5 Department of Endocrinology and Metabolism, Hospital Universitario Son Espases, 07120 Palma de Mallorca, Spain; 6 Centro de Investigación en Red de Enfermedades Respiratorias (CIBERES), Instituto de Salud Carlos III (ISCIII), 28029 Madrid, Spain; 7 Department of Pneumology, Hospital Universitario Son Espases, 07120 Palma de Mallorca, Spain; 8 Facultad de Medicina, Universidad de las Islas Baleares, 07120 Palma de Mallorca, Spain; 9 Department of Pediatrics, Hospital Universitario Son Espases, 07120 Palma de Mallorca, Spain; 10 Department of Clinical Analysis, Hospital Universitario Son Espases, 07120 Palma de Mallorca, Spain

**Keywords:** obstructive sleep apnea (OSA), pregnancy, oxidative stress, protein carbonyl, apnea–hypopnea index (AHI), gestational diabetes (GD), maternal–fetal health

## Abstract

Obstructive sleep apnea (OSA) is common during pregnancy and linked to adverse outcomes. While oxidative stress is a proposed pathogenic mechanism, evidence in pregnant populations remains limited. This multicenter, prospective study evaluated oxidative stress through protein carbonyl levels in 171 pregnant women and 86 cord blood samples. Polysomnography (PSG) performed during pregnancy categorized participants with the apnea–hypopnea index (AHI) in OSA, rapid eye movement (REM) OSA, and supine OSA. Protein carbonyl levels were measured by the dinitrophenyl hydrazine (DNPH) method. No significant differences were found in maternal or cord blood protein carbonyl levels between OSA and non-OSA groups, or between REM and supine OSA subgroups. Interestingly, women with shorter apnea–hypopnea (AH) length showed both higher maternal and cord blood protein carbonyl levels and lower nocturnal oxygen saturation. Overall, OSA in pregnancy was not associated with increased oxidative stress as measured by protein carbonyl levels. However, apnea–hypopnea duration and nocturnal hypoxia may influence oxidative stress, pointing to a complex relationship between OSA and oxidative stress during pregnancy, beyond traditional metrics like AHI. Future studies should explore additional biomarkers and diverse molecular pathways that could play a role, with special attention to emerging factors such as apnea–hypopnea length and hypoxic burden to elucidate the interrelationships between OSA and pregnancy more comprehensively.

## 1. Introduction

Obstructive sleep apnea (OSA), the most prevalent sleep-related breathing disorder, is characterized by recurrent episodes of complete (apnea) or partial (hypopnea) obstruction of airflow in the upper airway [1]. OSA induces arousal, elevated heart rate, blood pressure fluctuations, oxygen level variations, and systemic inflammation [2,3], all of which can adversely affect the course of pregnancy and fetal health [4].

The prevalence of OSA is notably elevated during pregnancy. It is reported to affect 11–30% in normal gestation and up to 70% of high-risk pregnancies [5,6]. Several physiological factors, including weight gain, hormonal fluctuations, and structural changes in the upper airway, have been implicated [7]. A growing body of research suggests that OSA during pregnancy is associated with an increased risk of preterm delivery, preeclampsia, gestational diabetes mellitus (GDM), and low birthweight in neonates [8,9]. However, there is considerable variability in reported prevalence rates [10,11,12,13,14] and conflicting evidence [15,16,17]. Remarkably, both the supine sleeping position and rapid eye movement (REM) sleep have been associated with greater OSA severity in the general population. REM-related OSA is particularly common in young women and has been independently linked to insulin resistance [18]. Further, REM apnea-hypopnea index (AHI) has shown significant associations with inflammatory biomarkers during pregnancy [2] and an increased risk of GDM [19].

Oxidative stress is recognized as a critical mechanism in numerous disorders, including cardiovascular disease, diabetes, and pregnancy complications [20]. OSA may increase oxidative stress, which can be improved when effective continuous positive airway pressure (CPAP) therapy reverses apnea [21]. Notably, OSA severity, particularly assessed by the oxygen desaturation index, has been found to correlate independently with oxidative stress [22].

Increments in oxidative stress have been proposed to be one of the main mechanisms associating OSA with adverse pregnancy outcomes [14]. Nevertheless, most evidence of this complex mechanistic pathway comes from non-pregnant populations, and it remains to be demonstrated whether it is also involved during pregnancy. Studies using animal models have demonstrated that intermittent hypoxia (IH) aggravates oxidative damage, promotes the formation of advanced glycation end products (AGEs), and disrupts placental function, thereby contributing to unfavorable pregnancy outcomes [23]. Moreover, a previous small study showed lower serum markers of oxidative and carbonyl stress in 23 OSA pregnant women compared to 41 women without OSA. However, these data are limited because OSA diagnosis was based on medical records (and not all of them during pregnancy), and no sleep studies were performed on the controls [24].

Oxidative stress arises from a dysregulation between heightened production of reactive oxygen species (ROS) and inadequate antioxidant defense mechanisms. This phenomenon leads to deleterious effects on lipids, proteins, and DNA [25]. The connection between OSA and oxidative stress is attributed to IH, which triggers a series of physiological reactions. This dynamic process impacts several biological pathways, contributing to the development and progression of complications associated with OSA.

Numerous techniques are available for quantifying oxidative stress. Indicators of oxidative stress encompass, among others, advanced oxidation protein products (AOPPs), which gauge protein damage [26]. Oxidative excess leads to the generation of AOPPs, which are oxidized proteins indicative of both oxidative stress and inflammation [27]. These AOPPs represent a more stable biomarker than products of lipid oxidation, underlining their significance in assessing the interplay between oxidative stress and inflammation [26].

Although much has been done in the last decade regarding the implications of OSA during pregnancy, much more has yet to be done in order to fully understand the clinical impact of OSA, as well as potential intermediate mechanisms such as oxidative stress, all of which may potentially help to identify patients at risk. Considering these findings, and to address these limitations, it is relevant to clarify the impact of OSA during pregnancy on oxidative stress. Our hypothesis suggests that pregnant women with OSA may exhibit changes in oxidative stress markers compared to pregnant controls. Therefore, we aimed to compare protein carbonyl concentrations of maternal and cord blood serum from pregnant women with and without OSA, as well as to examine the effect of additional OSA criteria (REM and supine OSA).

## 2. Results

### 2.1. Clinical Characteristics

A total of 171 pregnant women (85 with gestational diabetes mellitus (GDM)) were included. As shown in Table 1, women were divided into groups based on their apnea-hypopnea index (AHI), with 28 having OSA and 143 showing AHI < 5 h⁻^1^. Additionally, rapid eye movement (REM) OSA and supine OSA were found in 52 and 27 women, respectively (but supine AHI values were missing in 25 women). Women with AHI ≥ 5 h^−1^ were significantly older (*p* = 0.020). Moreover, pre-pregnancy body mass index (BMI) was higher in women with OSA than in the non-OSA group (*p* = 0.005). Similar significant differences in BMI were observed according to REM (*p* = 0.000) and supine OSA criteria (*p* = 0.009). Although no differences were detected in pre-gestational smoking, we did find more smokers during pregnancy in both the OSA and supine OSA groups. Maternal hemoglobin concentration was slightly higher in women with REM OSA than in the non-REM OSA group. As presented in Table 1, there were no significant differences in neck circumference, gestational age, first pregnancy prevalence, or glucose, sodium, potassium, total and LDL cholesterol, C—reactive protein (CRP) levels, or blood pressure based on several OSA criteria.

Table 2 shows the general characteristics, sleep symptoms, and parameters among participants. While total sleep time was similar across groups (approximately 432–437 min), those with AHI ≥ 5 h⁻^1^ experienced a slightly higher percentage of time spent in N3 sleep time. Frequent snoring and self-reported sleep time was found in greater proportion in women with supine OSA. No significant differences were found in the remaining OSA symptoms based on several criteria of OSA. As expected, patients with OSA had worse nocturnal oxygenation indices (minimum oxyhemoglobin saturation (SaO_2_), mean SaO_2_, desaturation index (DI), and CT90%) than non-apneic women.

AH length in the 50th percentile was 20.6 (16.1–29.3) s. There were no significant differences in the proportion of women who smoked during pregnancy according to the apnea-hypopnea (AH) length in the 50th percentile (10.1 vs. 9%, *p* = 0.81). However, mean SaO_2_ during the night was significantly lower when AH length *p* < 50 (96 (95–97) vs. 97 (95–97)%, respectively), and there was a tendency towards worse nocturnal SaO_2_ in other markers (minimum SaO_2_ = 92 (90–94) vs. 93 (90–95)%, *p* = 0.08; and DI = 0.5 (0–1.3) vs. 0.2 (0–0.7) h^−1^, *p* = 0.09).

### 2.2. Protein Carbonyl Concentration

**Figure 1:**
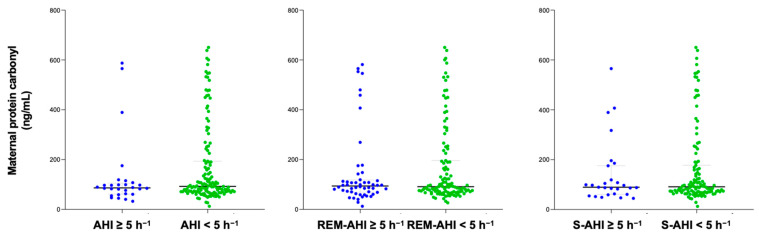
Maternal protein carbonyl content of pregnant women according to diverse OSA criteria. Blue color represents OSA samples and green non-OSA.

**Figure 2:**
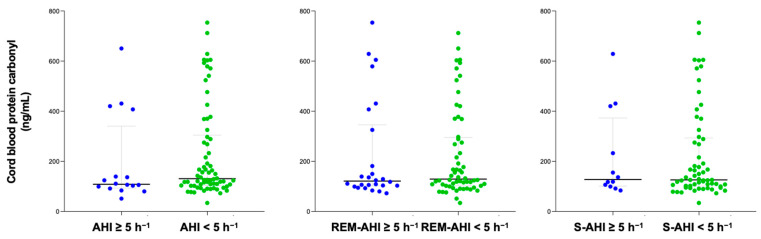
Cord blood protein carbonyl content according to diverse OSA criteria. Blue color represents OSA samples and green non-OSA.

The content of protein carbonyl was compared between groups with OSA and non-OSA, categorized according to their apnea–hypopnea index (AHI) and different sleep apnea criteria. The time between PSG and delivery in the study population was 31 (20–49) days. After delivery, 86 cord blood samples were collected to measure protein carbonyl levels. No significant differences were found in serum protein carbonyl levels in either pregnant women or in cord blood samples based on the presence of different sleep apnea criteria (Figure 1 and Figure 2). Similar findings were reported in a sub-analysis of both normal pregnancies and those with GDM, as well as in women who did not smoke.

Bivariate analysis showed significant correlations between maternal serum protein carbonyl concentration and maternal age (Rho = −0.26), time in REM (Rho = 0.16), and mean AH length (Rho = −0.25), while no relationships were found with cord serum protein carbonyl levels.

**Figure 3:**
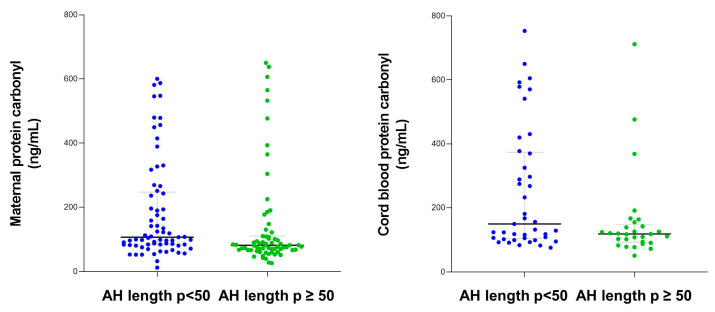
Maternal and cord blood protein carbonyl content according to apnea–hypopnea length.

Maternal and cord blood protein carbonyl levels were higher among women categorized into AH length *p* < 50 compared with women with AH length *p* ≥ 50 (Figure 3).

## 3. Discussion

Obstructive sleep apnea (OSA) is prevalent among pregnant women, and an association with adverse pregnancy outcomes such as preeclampsia and gestational diabetes mellitus (GDM) has been established [28]. However, the specific physio-pathological mechanisms promoted by OSA that lead to negative consequences remain unclear [14,29]. Oxidative stress has been proposed as a key mechanism that may contribute to maternal and fetal complications. Both OSA and GDM are known to increase oxidative stress through different yet potentially synergistic pathways [28,30]. The relationship between maternal health, oxidative stress, and long-term offspring outcomes is further underscored by recent studies investigating the predictors of OSA in young adults. A large retrospective study found that maternal conditions such as GDM, preeclampsia, and pregnancy-induced hypertension were associated with an increased risk of OSA in young adulthood. These conditions, which are known to elevate oxidative stress through the imbalance of reactive oxygen species (ROS) and impaired antioxidant defense systems, may predispose offspring to conditions like OSA later in life [31]. Additionally, maternal health factors such as obesity and preterm delivery have also emerged as predictors for OSA risk, further emphasizing the long-term effects of maternal metabolic health and oxidative stress on offspring [32]. Together, these findings reinforce the importance of managing oxidative stress during pregnancy in order not only to improve immediate pregnancy outcomes but also to reduce the risk of chronic conditions, such as OSA, in offspring later in life.

This study aimed to explore whether oxidative stress, as indicated by protein carbonyl levels, is elevated in pregnant women with OSA. No significant differences in protein carbonyl content were found between pregnant women with OSA and those without, nor in the cord blood samples of their newborns. This lack of difference persisted across various sleep apnea subtypes (OSA, rapid eye movement (REM) OSA, and supine OSA) and was consistent even when considering subgroups with and without GDM.

Protein carbonyl, as a marker of oxidative stress, has been extensively studied in the context of adverse pregnancy outcomes. Elevated protein carbonyl levels have been linked to conditions such as preeclampsia [33]. Similarly, protein carbonyl levels in GDM have been associated with insulin resistance and metabolic dysregulation, contributing to adverse fetal outcomes [34]. Further, an imbalance between ROS generation and antioxidant capacity appears to impair placental function, as evidenced by increased protein oxidation and lipid peroxidation markers in intrauterine growth restriction [35].

Oxidative stress is a well-established consequence in non-pregnant OSA populations and is primarily driven by intermittent hypoxia (IH), which promotes the overproduction of ROS and overwhelming antioxidant defenses, leading to oxidative damage in critical biomolecules, including proteins, lipids, and DNA. Protein carbonyls have been found to be consistently increased in patients with OSA [36,37]. This oxidative imbalance is involved in the pathogenesis of numerous conditions, such as cardiovascular disease [38], endothelial dysfunction [39], systemic inflammation [40], and pulmonary diseases, highlighting its critical role in the morbidity linked to OSA. The interplay between OSA and oxidative stress becomes more complex during pregnancy due to physiological adaptations that modulate oxidative responses. Animal models have provided valuable insights into the mechanisms underlying OSA-induced oxidative stress. Rodent studies, for instance, have shown that IH exacerbates oxidative damage, increases advanced glycation end products (AGEs), and impairs placental function, contributing to adverse pregnancy outcomes [23]. However, in most studies, only the hypoxic challenge is applied, which lacks a more realistic model that would include intermittent hypercapnia and sleep fragmentation, which could also influence oxidative stress. To the best of our knowledge, the impact of OSA on protein carbonyl in pregnant women has been previously analyzed only once: Khan et al. [24] included 74 pregnant women (23 with OSA). Unexpectedly, they found a higher protein carbonyl concentration in control subjects compared to women with OSA. Nonetheless, this study has important limitations that should be considered. In addition to having a relatively small sample size, the main limitation is that it was a retrospective study, in which the analysis was performed on women who had a previous diagnosis of OSA and who also had serum samples collected during pregnancy, but without information about the timing of the diagnosis of OSA in relation to pregnancy and gestational age. In addition, it was not exactly known how many of them were on continuous positive airway pressure (CPAP) treatment, what the compliance rates were, or whether they had lost weight after the diagnosis of OSA. Moreover, control women underwent no sleep study to confirm the absence of associated OSA, which was defined by no self-reported symptoms such as witnessed apnea, gasping, and snoring, which are known to have a poorly predictive value for diagnosis during pregnancy [41].

The lack of significant differences in protein carbonyl levels between the studied groups in the present study may be influenced by several factors. Although OSA in non-pregnant women is recognized for its role in promoting oxidative stress, physiological adaptations during pregnancy could attenuate this effect. Both pregnancy and OSA can affect oxidative and carbonyl stress levels [24,42,43]. Most of the women in the current study exhibited mild OSA. It is plausible that adaptive and preconditioning mechanisms are present in mild stages of OSA; however, these compensatory responses may be insufficient to provoke oxidative stress when individuals experience more severe levels of hypoxia and increased sleep fragmentation that surpass an as-yet undefined threshold. Therefore, pregnancy is associated with enhanced antioxidant defenses, which may help offset the oxidative stress generally associated with OSA. Notably, total antioxidant capacity increases significantly throughout pregnancy, peaking during labor at 37 to 41 weeks [44], a period characterized by the highest physiological stress. Another justification for our findings is that neonates were able to successfully adapt to OSA and IH during pregnancy. Indeed, evidence from an animal study showed that the placenta can mitigate fetal exposure to hypoxia resulting from maternal respiratory events. Specifically, fluctuations in oxygen levels within the umbilical vein were significantly less pronounced compared to those observed in maternal arterial blood following repeated obstructive apnea [45]. Finally, interindividual variations in protein carbonyl levels may result from the intricate interplay of oxidant and antioxidant mechanisms, influenced by factors such as genetic polymorphisms in oxidative metabolism, dietary intake, tobacco use, and lifestyle-related variables, including physical activity.

The results of this study may suggest that although OSA frequently coexists with pregnancy complications and oxidative stress is thought to play a role in such complications, OSA does not seem to directly contribute to these issues through oxidative stress mechanisms. Additionally, since our sample consisted of pregnant women and their corresponding cord blood samples, pregnancy status or stage is unlikely to have influenced our findings, as gestational age was accounted for in our analyses. Nonetheless, it is important to acknowledge that the participants in this study had mild OSA, which may affect the outcomes. Nevertheless, our results show higher protein carbonyl levels in women (and cord blood samples) with lower apnea-hypopnea (AH) length (AH length *p* < 50) during sleep. Attractively, this group of women had slightly worse nocturnal oxygenation parameters, suggesting that nocturnal hypoxia and length of obstructive events may act synergically to up-regulate oxidative stress during gestation. Currently, apnea-hypopnea index (AHI) alone (the classical parameter that defines OSA severity) is considered insufficient for the identification and explanation of OSA-associated health consequences. A new and promising parameter, the duration of respiratory events, has recently been described, with a better association with type 2 diabetes mellitus and mortality risk than AHI in OSA patients [46,47]. What is more, respiratory events are shorter in younger adults, women, non-REM sleep, and non-supine posture compared to older adults, men, REM sleep, and supine position, respectively. Given the current trend of emphasizing the importance of personalized sleep medicine, these results would encourage exploring the value of this new variable in the severity and consequences of OSA, particularly in pregnant women. Nonetheless, future studies are required to further explore the role of obstructive event duration and oxidative stress markers during pregnancy as well as their influence on hypothetical human gestation complications.

### Strengths and Limitations

The main strength of our study lies in its design, which enabled us to analyze protein carbonyl levels in both pregnant women and cord blood from samples of women, who were very well studied with the gold standard test for diagnosing OSA in the third trimester of pregnancy (polysomnography, PSG) in a multicenter, prospective study. Yet, this study has several potential limitations that should be considered. First, we measured only a single biomarker of oxidative stress—protein carbonyl—which may not fully reflect the extent of oxidative damage associated with OSA. Second, the relatively small sample size in the OSA group (n = 28) compared to controls (n = 143) and variability in OSA severity between participants may have reduced our ability to detect significant differences in oxidative stress levels. Third, relevant differences in age [48], body mass index (BMI) [49], and smoking [50] may have had an influence on the results, although it is probably minor, since patients with OSA were older and had both higher weight and smoking proportion during pregnancy, all of which are known to increase oxidative stress, and no differences were observed when women who had smoked were excluded from the analysis. Fourth, wide geographical variation exists in the prevalence of OSA [51]. We included mainly Caucasian women; thus, our results may not be applicable to different ethnic backgrounds. Fifth, supine AHI values were missing for 25 women, and this might have affected the results. Finally, our study population consisted of women with mild OSA with a low proportion of obesity; therefore, the results cannot be extrapolated to moderate–severe OSA with different characteristics (obese patients).

## 4. Materials and Methods

### 4.1. Study Population

Three Spanish university hospitals (Son Espases, Araba, and Miguel Servet) recruited pregnant women with both normal pregnancies and gestational diabetes mellitus (GDM), all with singleton pregnancies in their third trimester. Women with a history of chronic illnesses (pulmonary, cardiac, or kidney diseases), complicated pregnancies (gestational hypertension, preeclampsia, or any other major obstetrical complication), previous diagnosis of obstructive sleep apnea (OSA), or impending delivery because of maternal–fetal disease were not allowed to participate in the study. All individuals provided written informed permission, and the study was authorized by the Institutional Ethics Committee of the Balearic Islands (IB1510/10PI) [52].

### 4.2. Clinical and Sleep Evaluation

Anthropometric, clinical, and sleep-related information was gathered consistently through questionnaires and direct measurements. The data included age, body mass index (BMI), neck circumference, systolic and diastolic blood pressure, and tobacco use. Reports of subjective breathing issues, such as asphyxia episodes, snoring, bed partner-observed sleep pauses, nocturia, morning headaches, and morning fatigue, were recorded with two frequency options (never/sometimes and almost always/always). Additionally, subjective nighttime sleep duration and daytime sleepiness (measured by the Epworth Sleepiness Scale [ESS]) were documented over the previous four weeks.

Overnight-attended polysomnography (PSG) was performed on all women in the sleep laboratory and manually scored using conventional criteria [53]. The standard parameters for 30 s epochs were applied. Breathing was measured using nasal cannulas, oronasal thermistors, and thoracoabdominal stain gauges. At the same time, oxyhemoglobin saturation (SaO_2_) was measured using a pulse oximeter. The definition of apnea was established as a 90% drop in airflow that lasted for at least 10 s. A decrease in airflow (30–90%) for at least 10 s accompanied with a decline in SaO_2_ (≥3%) or final arousal was considered hypopnea. The apnea–hypopnea index (AHI) was calculated by dividing the total number of instances of apnea/hypopnea by the total number of hours of sleep. REM AHI and supine AHI were calculated as the ratio of apnea/hypopnea during REM and supine sleep to the total time in REM and in supine in hours, respectively. OSA was defined as an AHI of ≥5 h^−1^. Moreover, REM OSA and supine OSA were defined as a REM AHI and supine AHI of ≥5 h^−1^, respectively. MeanSaO_2_ during the night, minimum SaO_2_ (lowest values recorded during sleep), percentage of total study time spent with SaO_2_ < 90% (CT90%), and total number of drops in SaO_2_ ≥ 3% divided by total number of hours of sleep (desaturation index (DI)) were all calculated as indicators of nocturnal SaO_2_ [54].

### 4.3. Sample Acquisition and Preparation

The morning after the PSG, maternal blood samples were collected from all women in fasting conditions. Moreover, cord blood samples were collected immediately after delivery. Laboratory data included glucose, sodium, potassium, total and LDL cholesterol, and C-reactive protein (CRP). The remaining blood sample was centrifuged at 3000 rpm for 20 min to isolate the serum. The different serum aliquots were stored at −80 °C for further determination. Protein carbonyl levels were quantified on serum by the Protein Carbonyl Colorimetric Assay Kit (Tissue and Serum Samples), catalog number E-BC-K117-S (Elabscience Biotechnology Co., Ltd., Houston, TX, USA). The specific assay is based on the dinitrophenyl hydrazine (DNPH) method, where carbonyls react with DNPH to form derivatives that can be measured spectrophotometrically. The procedure was conducted according to the manufacturer’s protocol. Detection limits and sensitivity were 0.02–10 nmol/mgprot and 10 nmol/mgprot, respectively.

### 4.4. Statistical Analysis

Data are presented as mean ± standard deviation, median and interquartile range, or percentage. Differences between groups were analyzed using Student’s *t* test or the Mann–Whitney U test for continuous variables, and Fisher’s exact test (two-tailed) or the chi-square test for categorical variables. Spearman’s correlation was used to assess relationships between sleep parameters and general characteristics of pregnant women, and protein carbonyl levels. To analyze the effect of nocturnal apnea–hypopnea length on protein carbonyl, all included patients were categorized into two groups based on the 50th percentile of apnea–hypopnea (AH) length: (a) AH length percentile < 50% (*p* < 50); (b) AH length percentile ≥ 50% (*p* ≥ 50). Statistical software SPSS v.26 (IBM) was used and a two-sided *p* value of less than 0.05 was considered significant.

## 5. Conclusions

In summary, this study revealed that neither maternal nor cord blood protein carbonyl concentrations differed between obstructive sleep apnea (OSA) and non-OSA patients during pregnancy. However, maternal protein carbonyl concentrations were found to be negatively related to respiratory apnea–hypopnea (AH) length, representing the fact that OSA influence on oxidative stress during pregnancy could be more complex than its effect on apnea-hypopnea index (AHI) would imply.

The number of studies that have evaluated the interplay of OSA and pregnancy is still scarce, and the potential physio-pathological mechanisms by which OSA could contribute to poor pregnancy outcomes remains largely unknown. Further studies are warranted to explore diverse molecular pathways that could play a role. These should focus on expanding the panel of oxidative stress biomarkers to include markers such as 8-isoprostane or total antioxidant capacity. Additionally, longitudinal studies tracking oxidative stress from early pregnancy to postpartum, alongside fetal outcomes, with special attention to emerging factors such as AH length and hypoxic burden, would help to elucidate and characterize the potential complex interrelationships between OSA and pregnancy more comprehensively. Investigating the interplay between oxidative stress, inflammation, and metabolic dysfunction in this population is highly desirable and could also yield valuable insights.

## Figures and Tables

**Table 1:** General characteristics of pregnant women with and without OSA.

Variables		AHI ≥ 5 h^−1^ (n = 28)	AHI < 5 h^−1^ (n = 143)	REM AHI ≥ 5 h^−1^ (n = 52)	REM AHI < 5 h^−1^ (n = 119)	Supine ^ϕ^ AHI ≥ 5 h^−1^ (n = 27)	Supine ^ϕ^ AHI < 5 h^−1^ (n = 119)
Age (years)		36 (34–40)	34 * (32–37)	35 (33–39)	34 (32–37)	34 (33–37)	34 (32–38)
Gestational age at PSG (weeks)		36 (34–37)	34 (32–37)	34 (32–36)	35 (32–37)	35 (33–36)	34 (32–37)
BMI before pregnancy (Kg/m^2^)		26 (23–30)	23 * (21–26)	26 (23–29)	23 * (21–25)	26 (22–28)	23 * (21–25)
Weight gain from pre-pregnancy to PSG (kg)		8.1 (4.9–12.7)	10 (6.6–12.4)	8.4 (4.8–12)	10 (7–12.5)	8 (5.5–12.9)	10 (7–12)
Obese before pregnancy n (%)		6 (21.4)	11 (7.7)	11 (21.2)	6 (5)	4 (14.8)	7 (5.9)
First pregnancy n (%)		14 (51.9)	78 (54.5)	28 (54.9)	64 (53.8)	15 (57.7)	63 (52.9)
Pre-gestational smoker n (%)	No	14 (50.0)	81 (56.6)	23 (44.2)	72 (60.5)	14 (51.9)	71 (59.7)
	Yes	10 (35.7)	34 (23.8)	18 (34.6)	26 (21.8)	7 (25.9)	28 (23.5)
	Former smoker	4 (14.3)	28 (19.6)	11 (21.2)	21 (17.6)	6 (22.2)	20 (16.8)
Gestational smoker n (%)		7 (25)	9 * (6.3)	9 (17.3)	7 (5.9)	4 (14.8)	8 (6.7) *
Neck circumference (cm)		34 (33–37)	34 (32–35)	34 (32–36)	33.5 (32–35)	34 (32–36)	33 (32–35)
Waist-to-hip ratio		0.99 (0.94–1.00)	1.00 (0.97–1.05)	0.97 (0.93–1.01)	1.01 (0.97–1.04)	0.99 (0.91–1.03)	1.0 (0.97–1.05)
Glucose (mg/dL)		73 (68–78)	76 (71–81.5)	76 (70–82)	74 (70–80.5)	73 (71–78.5)	76 (71–81)
Sodium (mEq/L)		136 (135–138)	137 (136–138)	137 (136–138)	137 (136–138)	136 (136–139)	137 (136–138)
Potassium (mEq/L)		4.0 (3.9–4.1)	4.0 (3.9–4.2)	4.0 (3.8–4.2)	4.0 (3.9–4.2)	4 (3.9–4.2)	4 (3.9–4.2)
Total cholesterol (mg/dL)		260 (239.5–302.5)	259 (226–298)	259 (229.5–301.5)	259 (229–298.5)	255 (204.5–276)	259 (231–300)
LDL cholesterol (mg/dL)		153 (112.5–180.5)	152 (123–182)	153 (114–182)	151 (123–176)	144.5 (111.5–167.5)	155 (124–183)
CRP (mg/dL)		0.7 (0.3–1.7)	0.5 (0.2–1.9)	0.7 (0.3–1.4)	0.5 (0.2–1.9)	0.9 (0.3–2.7)	0.5 (0.2–1.9)
Hemoglobin (g/dL)		12.3 (11.8–12.9)	11.9 (11.3–12.6)	12.1 (11.7–12.9)	11.8 * (11.1–12.6)	12.1 (11.6–12.9)	11.9 (11.3–12.6)
Systolic BP (mmHg)		107 (102–117)	107 (100–114)	106 (100–116)	107 (101–114)	106 (101–114)	107 (100–115)
Diastolic BP (mmHg)		71 (62–75)	65 (60–71)	65 (62–74)	65 (59–72)	65 (61–74)	65 (60–72)

Descriptive analysis and comparison between groups. Continuous variables in mean ± SD if they followed a normal distribution or in median (IQR) if they followed a non-parametric distribution. For categorical variables n (%). * Significant differences (*p* < 0.05). Abbreviations: AHI, apnea–hypopnea index; BMI, body mass index; BP, blood pressure; cm, centimeter; CRP, C-reactive protein; dL, deciliter; g, grams; h, hours; h^−1^, per hour; HDL, high-density lipoprotein; HOMA-IR, homeostasis model assessment of insulin resistance; kg, kilograms; L, liter; m, meter; min, minutes; mg, milligrams; mmHg, millimeter of mercury; U, units; uL, microliter; PSG, polysomnography; QUICKI, qualitative insulin sensitivity check index. ^ϕ^ Supine OSA was determined in 146 women.

**Table 2:** Polysomnography data, and sleep symptoms in OSA and non-OSA groups.

Variables	AHI ≥ 5 h^−1^ (n = 28)	AHI < 5 h^−1^ (n = 143)	REM AHI ≥ 5 h^−1^ (n = 52)	REM AHI < 5 h^−1^ (n = 119)	Supine ^ϕ^ AHI ≥ 5 h^−1^ (n = 27)	Supine ^ϕ^ AHI < 5 h^−1^ (n = 119)
Total sleep time (min)	435 (416–460)	432 (409–457)	434 (410–457)	433 (410–456)	437 (413–457)	432 (409–458)
N1 + N2 sleep time (%)	57.6 (20–67.2)	62.8 (56.4–71)	60.9 (42.1–69.5)	63.2 (56.7–71.2)	62.3 (54.4–65.7)	64.9 (58.1–72.2)
N3 sleep time (%)	26.8 (20.9–65.4)	22.4 * (16.7–29.7)	26.8 (17.1–37.8)	22.8 (16.6–28.4)	23 (19–30.8)	21.2 (16.2–28)
REM sleep time (%)	11.4 (8.9–14.4)	13.1 (9.5–16.2)	12.7 (8.6–16.5)	13 (9.8–15.9)	13.2 (9–17.2)	13 (9.5–16)
AHI (h^−1^)	7.8 (5.9–10.1)	0.7 * (0.2–1.7)	3.3 (2–6.2)	0.6 ^¥^ (0.2–1.5)	7.7 (3.1–9.7)	0.7 ^¶^ (0.3–1.5)
Arousal index (h^−1^)	16.1 (4.9–22.8)	14.1 (2.1–20.7)	15.7 (9.9–22.8)	12.9 (0.6–20.7)	17 (2.6–22.8)	14.2 (1.4–22.1)
Mean SaO_2_ (%)	96 (95–97)	97 (95–97)	96 (95–97)	97 (95–97)	96 (95–97)	97 (95–97)
Minimum SaO_2_ (%)	90 (88–91)	93 (91–94)	91 (89–94)	93 (91–94)	91 (88–94)	93 (92–95)
CT90% SaO_2_ (min)	0 (0–0.4)	0 (0–0)	0 (0–0.2)	0 (0–0)	0 (0–0.3)	0 (0–0)
Desaturation index (h^−1^)	1 (0.3–2.5)	0.1 ^∞^ (0–0.6)	0.6 (0–1.8)	0.1 (0–0.6)	1.3 (0.4–2.8)	0 ^∞^ (0–0.5)
Nocturia n (%)	24 (85.7)	126 (88.1)	42 (80.8)	108 (90.8)	25 (92.6)	109 (91.6)
Reported apnea n (%)	5 (17.9)	16 (11.2)	4 (7.7)	17 (14.3)	6 (22.2)	10 (8.4)
Frequent gasping awakenings n (%)	8 (28.6)	26 (18.2)	10 (19.2)	24 (20.2)	8 (29.6)	23 (19.3)
Morning headaches n (%)	5 (17.9)	23 (16.1)	9 (17.3)	19 (16)	3 (11.1)	21 (17.6)
Unrefreshing sleep n (%)	17 (60.7)	87 (60.8)	34 (65.4)	70 (58.8)	16 (59.3)	73 (61.3)
Snoring (always/frequent) n (%)	22 (78.6)	77 (53.8)	33 (63.5)	66 (55.5)	22 (81.5)	60 (50.4) ^∞^
Reported sleep time, working days (h)	7.3 (6–8.5)	7 (6.5–8)	7.5 (6–8)	7 (6.5–8)	8 (7–8)	7 (6.5–8) ^∞^
Reported sleep time, weekends (h)	8 (6–9)	8 (7–8.5)	8 (7–9)	8 (7–8)	8.5 (7.5–9)	7.5 (6.5–8.5) ^∞^
Epworth Sleepiness Scale	7 (5–9)	6 (4–8)	6 (4–9)	7 (5–8)	7 (4–8)	6 (4–8)

Continuous variables in mean ± SD if they followed a normal distribution or in median (IQR) if they followed a non-parametric distribution. For categorical variables n (%). * Significant differences (*p* < 0.05) between AHI ≥ 5 h^−1^ and AHI < 5 h^−1^; ^¥^ significant differences (*p* < 0.05) between REM AHI ≥ 5 h^−1^ and REM AHI < 5 h^−1^; ^¶^ significant differences (*p* < 0.05) between supine AHI ≥ 5 h^−1^ and supine AHI < 5 h^−1^; ∞ significant differences (*p* < 0.05) between desaturation index > 1 h^−1^ and desaturation index < 1 h^−1^. Abbreviations: AHI, apnea–hypopnea index; CT90%, sleep time with oxygen saturation <90%; h, hours; h^−1^, per hour; min, minutes; N1 + N2, superficial sleep time; N3, deep sleep time; OAI, obstructive apnea index; REM, rapid eye movement; SaO_2_, oxygen saturation. ^ϕ^ Supine OSA was determined in 146 women.

## Data Availability

Data cannot be made publicly available for ethical reasons, as the study participants did not give consent for public data sharing: due to the ethical restrictions related to the consent of the participants of the public deposition of the data, as well as legal limitations of the Spanish legislation on data protection, it is not possible to obtain permission to publicly access the database. However, other researchers who meet the criteria for access to confidential data may apply for access to the minimal dataset underlying the results upon request to the Ethics Committee (ceic_ib@caib.es), which will evaluate the data request proposal within the meaning of medical research involving human subjects.

## Abbreviations

OSA	obstructive sleep apnea
AHI	apnea–hypopnea index
DNPH	dinitrophenyl hydrazine
AH	apnea–hypopnea
GDM	gestational diabetes mellitus
REM	rapid eye movement
CPAP	continuous positive airway pressure
IH	intermittent hypoxia
AGEs	advanced glycation end products
ROS	reactive oxygen species
AOPPs	advanced oxidation protein products
IQR	interquartile range
BMI	body mass index
BP	blood pressure
CRP	C-reactive protein
PSG	polysomnography
QUICKI	qualitative insulin sensitivity check index
HOMA-IR	homeostasis model assessment of insulin resistance
HDL	high-density lipoprotein
cm	centimeter
dL	deciliter
g	grams
h	hours
h^−1^	per hour
kg	kilograms
L	liter
m	meter
min	minutes
mg	milligrams
U	units
mmHg	millimeter of mercury
uL	microliter
CT90%	sleep time with oxygen saturation <90%
N1 + N2	superficial sleep time
N3	deep sleep time
OAI	obstructive apnea index
DI	desaturation index
ESS	Epworth Sleepiness Scale
PSG	polysomnography
SaO_2_	oxygen saturation
